# Exploring the Potential of PLGA Nanoparticles for Enhancing Pulmonary Drug Delivery

**DOI:** 10.1021/acs.molpharmaceut.5c00118

**Published:** 2025-06-06

**Authors:** Mirsiane Pascoal Costa, João Octavio Carneiro Abdu, Maria Fernanda Cobucci Soares de Moura, Allana Carvalho Silva, Thiago Medeiros Zacaron, Mayara Rodrigues Brandão de Paiva, Rodrigo Luiz Fabri, Frederico Pittella, Ítalo Tuler Perrone, Guilherme Diniz Tavares

**Affiliations:** † Postgraduate Program in Pharmaceutical Science, 28113 Federal University of Juiz de Fora, Juiz de Fora 36036-900, Minas Gerais, Brazil; ‡ Faculty of Pharmacy, Federal University of Juiz de Fora, Juiz de Fora 36036-900, Minas Gerais, Brazil; § Department of Biochemistry, Institute of Biological Sciences, Federal University of Juiz de Fora, Juiz de Fora 36036-900, Minas Gerais, Brazil

**Keywords:** lung diseases, PLGA, nanoparticles, pulmonary delivery, nanotechnology

## Abstract

Lung diseases remain a leading cause of mortality globally, posing a substantial challenge to public health. Conditions such as asthma, tuberculosis, cystic fibrosis, pneumonia, chronic obstructive pulmonary disease (COPD), and lung cancer are highly prevalent and of increasing concern due to their rising incidence in recent years. The recent global outbreak of coronavirus disease 2019 (COVID-19) has further highlighted the urgent need for more effective therapeutic approaches to combat pulmonary diseases. In this context, growing interest in nanotechnology for pulmonary drug delivery has emerged, driven by its potential to enable localized treatment, reduce dosages, provide controlled release, enhance drug solubility, and improve bioavailability. Among the various nanomaterials explored, poly­(lactic-*co*-glycolic acid) (PLGA)—a copolymer of lactic and glycolic acids—has gained regulatory approval as a safe, biodegradable, and biocompatible carrier, with an extended-release profile, making it an ideal candidate for the development of nanostructured drug delivery systems. Multiple methodologies are available for synthesizing PLGA nanoparticles tailored to pulmonary administration, supported by a wide array of devices designed to cater to individual patient needs. This review seeks to evaluate the advantages of PLGA-based nanoparticles for pulmonary drug delivery, with a focus on their potential to enhance inhalation therapy formulations.

## Graphical abstract

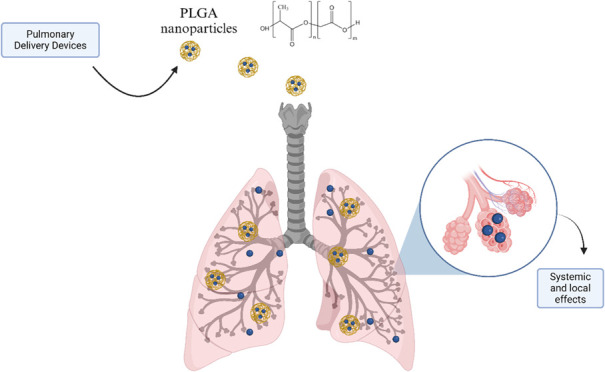

## 1 Introduction

The lungs have long been recognized as a key target for the treatment of various pulmonary disorders, including chronic obstructive pulmonary disease (COPD), lung cancer, asthma, infections such as tuberculosis (TB), cystic fibrosis (CF), pneumonia, and idiopathic pulmonary fibrosis (IPF). Many of these conditions are severely debilitating and, in certain cases, life-threatening. Despite advancements in therapeutic approaches, no current treatment has demonstrated the ability to fully restore lung function.^1^

Respiratory diseases primarily impact the trachea, bronchi, lungs, and thoracic region. Patients with mild cases typically exhibit symptoms such as cough, chest pain, and breathing impairment. In more severe cases, the condition may escalate to respiratory distress, a sensation of oxygen deprivation, respiratory failure, and, in some instances, death.^2^ Respiratory diseases have historically represented a major public health concern. According to data from the World Health Organization (WHO), chronic obstructive pulmonary disease (COPD), lower respiratory tract infections, and lung cancer are consistently ranked among the top ten leading causes of global mortality.^3^ Moreover, following the COVID-19 pandemic in 2019, there has been a significant increase in the number of patients with respiratory diseases, presenting a considerable global threat.^4,5^ In light of this, the quest for more effective therapies for the treatment of these conditions has been extensively explored, with a particular emphasis on the research and development of novel drugs for pulmonary administration.^6–14^

Pulmonary drug administration is a pharmacological targeting strategy that can facilitate both local action within the lungs and systemic effects.^15^ For locally acting drugs, pulmonary administration allows for the delivery of lower doses directly to the target site. This not only reduces systemic exposure and minimizes adverse effects but also, in some cases, facilitates a rapid onset of action.^16^ In the case of systemically acting drugs, this route offers the advantage of bypassing injections, particularly for compounds with poor gastrointestinal absorption, while also promoting more favorable pharmacokinetic profiles.^17,18^ Despite the aforementioned advantages, pulmonary administration is relatively complex, primarily due to the respiratory tract’s defense mechanisms, which are designed to prevent inhaled materials from entering the lungs and to remove or inactivate them following deposition.^17^ To overcome these challenges, nanostructured delivery systems present effective alternatives, as their small size enables them to evade pulmonary clearance and enhance absorption.^19,20^

In this context, nanoparticles (NPs) composed of PLGA (poly­(lactic-*co*-glycolic acid)) are of particular interest due to their biodegradability, biocompatibility^21,22^ low toxicity, modified release characteristics, and ability to protect encapsulated drugs from degradation.^23,24^ Consequently, recent years have seen numerous studies published and patents registered concerning the use of these NPs for pulmonary administration.^25–34^

Considering the above, this review aims to underscore the potential of PLGA NPs as highly effective delivery systems for pulmonary drug administration. This review will explore in detail the application of PLGA NPs in pulmonary pharmaceutical formulations, while also addressing the challenges and opportunities associated with the inhalation route in the treatment of respiratory diseases. The review was conducted through a comprehensive analysis of the Google Scholar, PubMed, ScienceDirect, Web of Science, and Espacenet databases, encompassing publications from 2014 to 2024

## 2 Pulmonary Route: Challenges and Future Directions in Drug Delivery

The pulmonary route of administration exhibits distinctive features that make it a highly attractive strategy for both local and systemic drug delivery. As a noninvasive and painless alternative to intravenous and intramuscular routes, it facilitates self-administration and contributes to improved patient adherence to therapy.^35^ Moreover, this route is particularly advantageous for the delivery of drugs with poor oral bioavailability or those prone to degradation by gastric acid and hepatic first-pass metabolism. In addition, pulmonary administration allows for targeted delivery to specific regions of the lungs, thereby enhancing therapeutic efficacy while reducing systemic side effects.^36^

The respiratory membrane, consisting of the alveolar epithelium and the capillary endothelium (Figure 1), has an approximate thickness of 0.5 to 1.0 μm and is highly vascularized, providing high permeability and enabling rapid drug absorption.^35,36^ Moreover, the low enzymatic activity and the absence of first-pass hepatic metabolism further reduce drug degradation.^37^ As a result, the large surface area of the lungs—estimated at approximately 100 m^2^—combined with these physiological advantages, supports efficient drug delivery and allows for reduced dosing compared to conventional administration routes.^38^

**Figure 1:**
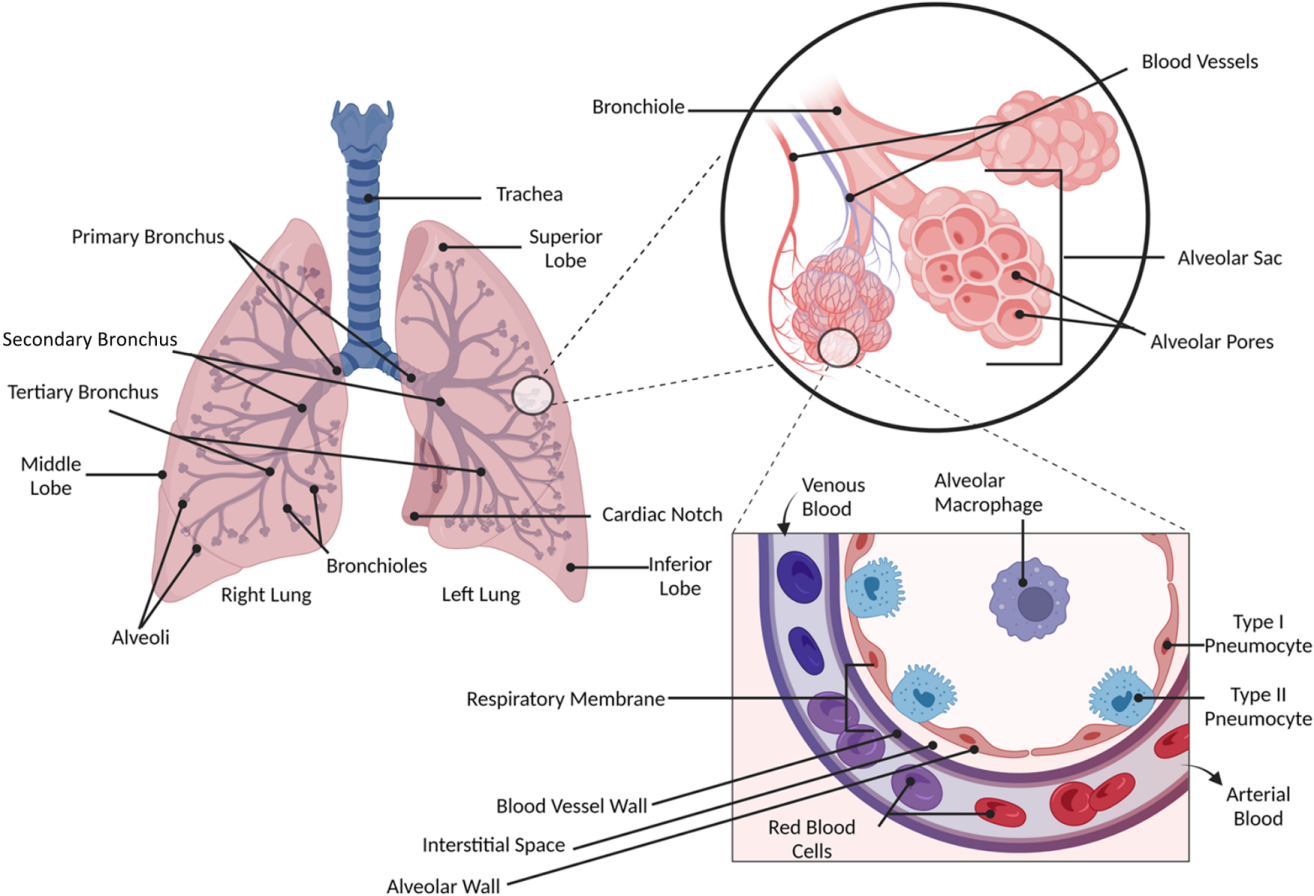
Schematic illustration of the human respiratory system places particular emphasis on the pulmonary and alveolar structure. On the left, the macroscopic anatomy of the lungs is illustrated, displaying the bronchial tree, which is comprised of the trachea, primary, secondary, and tertiary bronchi, as well as the bronchioles that diverge into the alveoli. On the right, a magnification of the microscopic structure of the alveoli, where gas exchange occurs. The alveolar epithelium is composed of two distinct types of pneumocytes: type I and type II. Type I pneumocytes form the alveolocapillary barrier, which is responsible for facilitating gas exchange, while type II pneumocytes synthesize pulmonary surfactant, a crucial component in maintaining optimal lung function. Additionally, alveolar macrophages are visible, which are phagocytic cells that play a pivotal role in lung defense, eliminating inhaled particles and microorganisms. In the expansion of the alveolocapillary barrier, the respiratory membrane is particularly notable, comprising the alveolar wall, the interstitial space, and the wall of the blood capillaries.

Despite the numerous advantages offered by the pulmonary route, several challenges can compromise the therapeutic efficacy of drugs delivered via this pathway.^39–41^ Among these, particle size plays a pivotal role in determining the extent of lung penetration. Nanoparticles (NPs) with diameters around 200 nm face significant barriers to diffusion, as their size closely approximates the estimated pore size of human airway mucus. As a result, the mucus gel layer acts as a “sticky net”, effectively trapping inhaled NPs of similar dimensions and impeding their transport through the airway barrier.^42^

For optimal pulmonary drug delivery, particles with an aerodynamic diameter between 1 and 5 μm are more likely to reach the deeper regions of the lungs. In contrast, particles with aerodynamic diameters greater than 10 μm are typically unable to penetrate beyond the upper airways due to their size, thereby limiting their ability to reach the intended site of action.^43^

In the lung epithelium, type II pneumocytes secrete pulmonary surfactant, a complex mixture of phospholipids and surfactant-associated proteins. These proteins play a critical role in innate alveolar defense by facilitating the adhesion and agglomeration of foreign particles, which are then cleared by ciliated epithelial cells or alveolar macrophages. While essential for host protection, this mechanism may also affect inhaled therapeutics by promoting their removal from the alveolar surface prior to absorption,^44^ as previously reported in the literature.^6^

Pulmonary macrophages play a central role in the clearance of inhaled particles through phagocytosis. It is estimated that each of the approximately 500 million alveoli in the human lungs harbors around 12 to 14 macrophages, which actively internalize foreign particles ranging from 0.5 to 5.0 μm that reach the lower respiratory tract—including not only pathogens but also therapeutic agents.^38,45^ This robust phagocytic activity presents a significant barrier to pulmonary drug delivery, as it can reduce drug retention at the target site and compromise therapeutic efficacy.

To overcome the aforementioned challenges, numerous studies have explored the use of poly­(lactic-*co*-glycolic acid) (PLGA) NPs to improve the efficacy of pulmonary drug delivery. In this context, Bahlool et al.^46^ developed inhalable PLGA nanoparticles encapsulating all-trans-retinoic acid (ATRA) for the treatment of tuberculosis via host-directed therapy, specifically targeting human macrophages infected with . The clinical utility of ATRA is limited by its low aqueous solubility and short half-life, which hinder the attainment of therapeutic concentrations at the site of infection and contribute to systemic toxicity. Encapsulation of ATRA within PLGA NPs for pulmonary delivery enhances macrophage targeting, facilitates drug accumulation in the lungs, and reduces off-target effects, thereby optimizing therapeutic outcomes.

### 2.1 Devices for Pulmonary Delivery

Currently, inhalation therapy is regarded as the optimal alternative for treating pulmonary diseases such as chronic obstructive pulmonary disease (COPD), asthma, and cystic fibrosis (CF) The primary devices utilized in inhalation therapies include nebulizers (Figure 2A), pressurized metered-dose inhalers (pMDIs) (Figure 2B), soft mist inhalers (SMI) (Figure 2C), and dry powder inhalers (DPIs) (Figure 2D).^47–50^ The selection of the most suitable device must consider the specific drug, formulation, and the patient’s condition.

**Figure 2:**
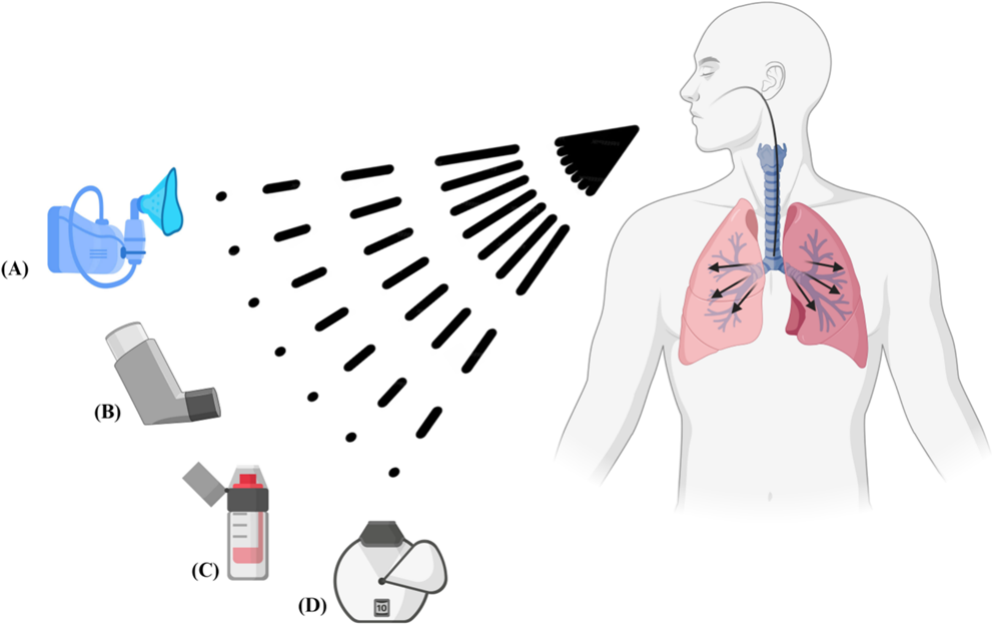
Inhalation formulations can be administered using a variety of devices, including (A) nebulizers, (B) pressurized metered-dose inhalers (pMDIs), (C) soft mist inhalers (SMIs), and (D) dry powder inhalers (DPIs), all of which are employed for pulmonary drug delivery.

Different inhalation devices influence aerosolization performance in distinct ways. Therefore, selecting an appropriate device for inhalable nanoformulations requires thorough evaluation of aerosolization parameters to determine the most suitable option. Key parameters such as fine particle dose (FPD), fine particle fraction (FPF), mass median aerodynamic diameter (MMAD), geometric standard deviation (GSD), drug deposition, delivery rate, total delivery amount, and dose uniformity must be assessed when examining the compatibility of an inhalation device with a specific formulation. Ideally, the chosen device should ensure a consistent and appropriate aerodynamic diameter, efficient lung deposition, rapid drug delivery to the target site, and precise dosing to achieve optimal therapeutic outcomes.^51^

An overview of each delivery device, along with its advantages and disadvantages, is provided below.

#### 2.1.1 Nebulizers

Multiple types of nebulizers are available on the market, with jet nebulizers being the most commonly used in clinical practice. In this model, the collision between a liquid and a high-speed gas jet generates aerosol particles within the nebulizer chamber. Despite their widespread use, treatment with this type of device can be lengthy, and the air compressor is often heavy and noisy. Furthermore, the mechanical forces involved in aerosol generation may impact the stability and efficacy of certain drugs.^52^

Ultrasonic nebulizers are preferred among the various models due to their higher aerosol output capacity compared to jet nebulizers. This model employs a rapidly vibrating piezoelectric crystal to generate aerosol particles. The ultrasonic vibrations produced by the crystal are transmitted to the surface of the drug solution, forming stationary waves. Ultimately, droplets detach from the crests of these waves and are released as an aerosol.^52,53^ In contrast to jet nebulizers, ultrasonic models operate silently and provide faster nebulization. However, they are not suitable for nebulizing high-viscosity liquids or suspensions, and the piezoelectric crystal can heat the liquid in the reservoir, rendering the device inappropriate for thermosensitive drugs.^52–54^

Vibrating mesh nebulizers are capable of aerosolizing liquids or suspensions, functioning in either active or passive mode. In active mode, an aperture plate vibrates at a high frequency, drawing the solution through the apertures. Conversely, in passive mode, the mesh is connected to a transducer horn that transmits the vibrations from the piezoelectric crystal, forcing the solution through the mesh to generate the aerosol.^55^ Compared to other nebulizer models, mesh nebulizers offer greater precision, efficiency, and consistency in drug administration, while also being silent and generally portable.^56,57^

Generally, since no specific coordination is required from the patient to inhale the medication in aerosol form, children and patients on ventilation can effectively utilize this device. However, its operation relies on a power supply, which limits its practicality for everyday use.^49^

For nebulized nanoformulations, the excipient requirements are minimal; apart from nanocarrier components, only suspending aids are typically included, which have negligible impact on the nebulization process.^51^

Moreover, nanosuspensions prepared for nebulization benefit from the availability of various preparation methods, offering flexibility in formulation development. Compared to conventional formulations, nanosuspensions are easier to nebulize and demonstrate superior lung deposition rates. This is primarily because the aggregates of nanoformulations in droplets exhibit optimal aerodynamic diameters, enhancing their deposition efficiency in pulmonary tissues.^51,58^

#### 2.1.2 Pressurized Metered-Dose Inhalers (pMDIs)

The first portable multidose devices designed for inhalation, pressurized metered-dose inhalers (pMDIs), facilitate the administration of single or combined drugs.^50,59^ Typically, this device comprises a metal container that houses the drug in solution or suspension, filled with liquefied propellant gas under pressure; a dosing valve that enables the precise dispensing of the drug; and a plastic nozzle that allows the patient to activate the device, facilitating the entry of particles into the airways. However, its use can be challenging due to the requirements for preparation, shaking before use, synchronization between device activation and inhalation, as well as maintaining a constant and rhythmic inhalation followed by breath-holding.^59–62^

Several factors suggest that pMDIs may not be optimal for delivering inhalable nanoformulations to the lungs. First, the coordination required between actuation and inhalation is challenging, leading to significant variability in lung deposition rates and making precise dosing of nanoformulations difficult.^63–65^ Second, the hydrofluoroalkane (HFA) propellants commonly used in pMDIs can dissolve certain nanoformulations, potentially disrupting their nanostructures and compromising their stability. High shear stress during aerosol release further risks structural disruption, which may result in the leakage of the drug and reduce the drug stability. This instability can decrease bioavailability and hinder the therapeutic efficacy of the formulation.^51,64^

Additionally, ethanol, often employed as a cosolvent to stabilize nanosuspensions in MDIs, can alter aerosol performance. The selection of propellants and cosolvents also influences evaporation kinetics, impacting delivery efficiency. Lastly, in cases involving large-dose nanoformulations, flocculation may occur, reducing drug delivery accuracy and consistency.

#### 2.1.3 Soft Mist Inhalers (SMIs)

SMIs are portable, propellant-free devices that utilize mechanical energy to rapidly deliver single or multiple aerosol doses of inhalable drugs into the airways.^60,66^ Upon activation, a measured dose of the drug in solution is atomized and dispensed as two fine jets that converge at a predefined angle. This collision creates a cloud of inhalable particles, which patients can absorb by inhaling deeply and slowly. The aerosol cloud generated lasts approximately 1.5 s, facilitating synchronization between cloud formation and the patient’s inhalation.^50,67^ Additionally, SMIs exhibit low deposition in the oropharyngeal region^66,68^ and do not utilize propellants.^60^ However, despite the advantages of easier administration due to the prolonged duration of the aerosol cloud, SMIs still require coordination between cloud formation and inhalation.^60^

#### 2.1.4 Dry Powder Inhalers (DPIs)

DPIs are portable and compact devices that generate drug aerosols through patient inhalation. They contain a mixture of micronized drug particles transported by larger carrier molecules, most commonly lactose.^50^ These devices were designed to eliminate the need for propellant liquids and to simplify the formulation of insoluble therapeutic agents.^60^ Effective use of DPIs requires relatively high inspiratory flow rates to ensure proper dispersion of the powder and accurate drug delivery.^69–71^ For this reason, these devices may be less effective for patients with compromised lung function, such as those with chronic obstructive pulmonary disease (COPD).^69^

The primary considerations for dry powder inhalers (DPIs) when used with nanoformulations include: (i) evaluating whether the drying process impacts the integrity or stability of the nanoformulation; (ii) determining how the nanoformulation transitions into a solid microscale powder and assessing its efficiency in achieving effective lung deposition; and (iii) ensuring that the deposited solid microscale powder can redisperse back into its original nanoformulated state within the lung environment.^51^

## 3 PLGA: A Versatile Polymer for Pharmaceutical Application

Poly­(lactic-*co*-glycolic acid) (PLGA) is a copolymer composed of poly­(lactic acid) (PLA) and poly­(glycolic acid) (PGA).^24,72,73^ It has diverse applications in drug delivery systems, surgical and medical devices, and tissue engineering. This biomaterial is characterized by its biocompatibility, biodegradability, and low toxicity^74^ as well as its sustained release properties in biological environments.^24^ Thus, its use is approved by health regulatory agencies such as the FDA and EMA.^72,75^ Furthermore, PLGA undergoes hydrolysis into its monomers, lactic acid and glycolic acid. These monomers are endogenous to the body and are metabolized mainly via the Krebs cycle,^24,76,77^ followed by excretion via the lungs and urine,^78^ thereby reinforcing its safety profile.

PLGA synthesis occurs through two primary approaches: direct polycondensation of cyclic lactides and glycolides and ring-opening polymerization (ROP) of lactides and glycolides.^79^ Polycondensation involves polymerizing monomers under stirring and melting conditions, with or without a catalyst, but it often yields low molecular weight copolymers due to challenges in water removal during synthesis. This limitation, coupled with its economic disadvantages for producing high molecular weight PLGA, has reduced its applicability.^80^ Conversely, ROP is preferred for producing high molecular weight PLGA, utilizing tin-based catalysts, such as tin­(II) 2-ethylhexanoate (Sn­(Oct)­2), which offer cost-efficiency and high catalytic activity at elevated temperatures. For biomedical use, residual tin levels must be below 20 ppm, achieved through minimal catalyst usage or solvent-based purification. Low catalyst concentrations are standard in synthesizing high-glycolide PLGA due to its insolubility in most organic solvents.^81^ For a more comprehensive examination of PLGA synthesis, please refer to the cited literature.^79–81^

The physicochemical properties of PLGA render it one of the most attractive polymers within the biocompatible and biodegradable category.^74^ The molar ratio of its individual monomer components—lactic acid (LA) and glycolic acid (GA)—significantly influences its physical characteristics, including melting temperature, solubility, degree of crystallinity, mechanical strength, swelling, and hydrolysis capacity.^80^ Consequently, these factors affect the rate of drug release from the polymeric matrix. PLGA 50:50, consisting of 50% LA and 50% GA, is the most commonly utilized formulation for drug delivery.^82^ This ratio yields a highly hydrophilic and amorphous polymer with a rapid degradation rate, facilitating faster drug release from nanoparticles.^75,76,83^ Conversely, an increase in the proportion of LA enhances the polymer’s hydrophobicity and degradation resistance due to the presence of the methyl side chain in lactic acid units.^73^ This variability in the LA/GA ratio enables the production of polymers with a diverse array of properties, including varying melting temperatures and solubility in several organic solvents, such as tetrahydrofuran, dichloromethane, chloroform, acetone, and benzyl alcohol.^75,79^

PLGA copolymers with a higher GA content exhibit increased hydrophilicity, resulting in greater water absorption and accelerated degradation, which can be beneficial for the controlled and sustained release of therapeutic agents.^75,76^ On the other hand, polymers with a higher LA content are more hydrophobic, absorb less water, and degrade at a slower rate.^24^ The ratio of monomers in PLGA also affects encapsulation efficiency and drug release kinetics, with low molecular weight PLGA demonstrating faster degradation rates.^75,76,80^

The optically active poly­(d-lactic acid) (PDLA) and poly­(l-lactic acid) (PLLA) are similar in terms of their physicochemical properties. However, due to the structural differences in the polymer chains, PLA can exist in either a crystalline form (PLLA) or an amorphous form (PDLA), depending on the arrangement of the chains. Thus, PLGA with a higher proportion of l-lactide tends to have increased crystallinity, which results in slower degradation and a more sustained drug release profile. In contrast, PLGA with a higher proportion of d-lactide is more amorphous, leading to faster degradation and a more rapid drug release.^22,84^

The drug release from PLGA particles typically occurs through diffusion and/or uniform bulk erosion of the biopolymer matrix. The diffusion rate is largely determined by the drug’s diffusivity and partition coefficient, which are, in turn, influenced by the physicochemical properties of the drug, such as molecular size, hydrophilicity, and charge. A higher content of a water-soluble drug encourages water infiltration into particles, leading to the formation of a highly porous polymer structure as the drug is leached out. Conversely, hydrophobic drugs can restrict water diffusion into the microparticle system, thereby slowing the rate of polymer degradation.^85,86^

The terminal group of PLGA, whether it is an acid or an ester, significantly influences its hydrophilicity and degradation rate, with ester-terminated PLGA exhibiting enhanced resistance to hydrolysis.^76^ The interplay of these factors facilitates the tailoring of PLGA properties for various applications, ranging from drug release systems to the fabrication of diverse structures, such as microspheres and nanoparticles.^75,80,87^

Recent years have witnessed a surge in the development of nanoparticles composed of PLGA, attributed to their notable advantages, including biodegradability, biocompatibility, low toxicity, rapid degradation, protection of encapsulated drugs, and the capacity to modulate sustained release while targeting specific tissues.^22^ A substantial body of research has emerged focusing on the application of these nanoparticles for pulmonary drug delivery, aimed at treating various pathologies, including asthma, cystic fibrosis, pulmonary fibrosis, lung cancer, and tuberculosis.^6–14^

## 4 Synthesis of PLGA-Based Nanoparticles

PLGA can be utilized in a nanostructured form for the encapsulation of a wide range of materials, including hydrophilic and hydrophobic drugs, proteins, peptides, and macromolecules.^77,88^ The biocompatibility and biodegradability of PLGA NPs reflect the intrinsic properties of the polymer, as demonstrated by numerous studies.^89–94^ Therefore, it is safe as its products are metabolized in the body and eliminated through the lungs and urine,^78^ as shown in Figure 3.

**Figure 3:**
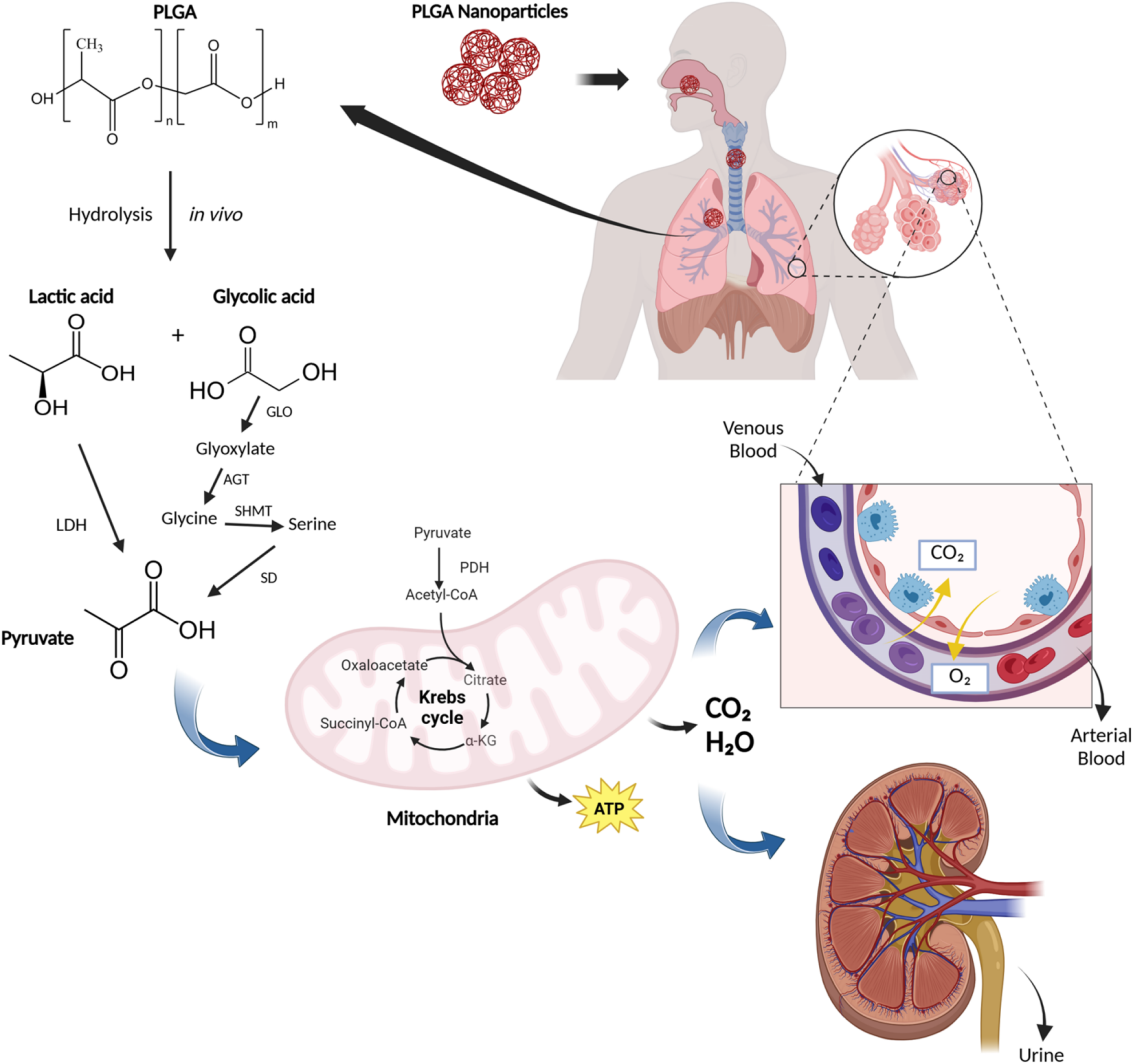
In vivo hydrolysis of PLGA-based nanoparticles occurs in the extracellular environment. The polymer is broken down into lactic and glycolic acids. The former is converted to pyruvate by the action of lactate dehydrogenase (LDH), while the latter is first converted to glyoxylate by glyoxylate oxidase (GLO), then to glycine by glyoxylate aminotransferase (AGT), which is then converted to serine by serine hydroxymethyltransferase (SHMT). Finally, serine is converted to pyruvate by serine dehydratase (SD). Pyruvate is then converted to acetyl-CoA by pyruvate dehydrogenase (PDH). Finally, the product formed is converted to carbon dioxide and water via the Krebs cycle and excreted via urine and lungs.

Various preparation methods have been employed, with the solvent emulsification-evaporation technique being the most commonly used, both in its single and double emulsion variants.^88,95^ Other prevalent processes for the synthesis of PLGA nanoparticles include salting out, nanoprecipitation, solvent emulsification-diffusion, spray-drying, and dialysis.^46,73,76^ Representative scanning electron microscopy (SEM) images, presented in Figure 4, illustrate the morphological characteristics of PLGA nanoparticles synthesized using selected preparation methods discussed in the text.

**Figure 4:**
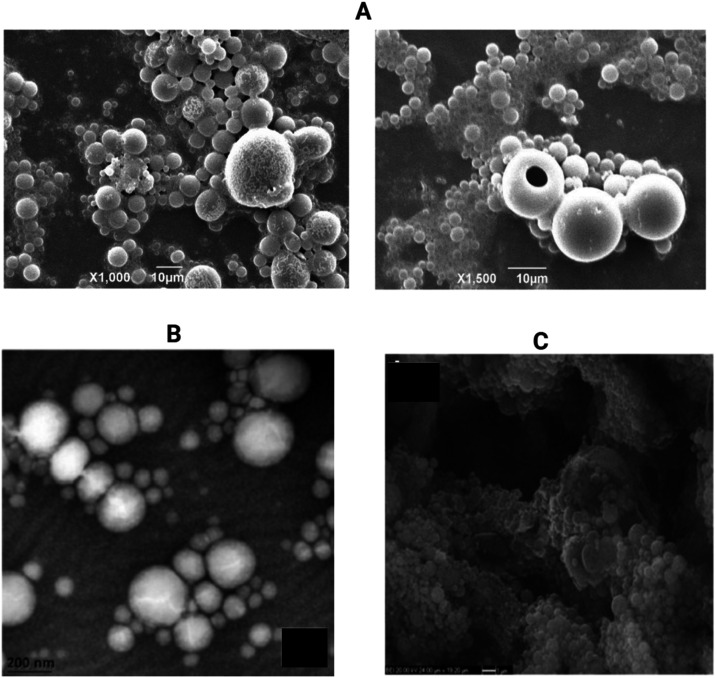
Representative SEM micrographs of PLGA NPs produced via various synthesis technique. (A) PLGA NPs (left) and PLGA NPs coated with chitosan (right) obtained using the single emulsion-solvent evaporation method (reproduced with permission from ref 107; Copyright 2025 Elsevier); (B) PLGA NPs synthesized via the nanoprecipitation method (reproduced with permission from ref 108; Copyright 2017 Elsevier); (C) PLGA NPs prepared by the double emulsion-solvent evaporation technique adapted from ref 89. Available under a CC-BY 4.0. Copyright 2015 Springer Nature.

### 4.1 Emulsification

Emulsification is the predominant method employed for the preparation of PLGA nanoparticles, primarily due to the simplicity of the process.^96^ The selection of this method is based on the specific characteristics of the molecule intended for encapsulation.

#### 4.1.1 Simple Emulsion-Solvent Evaporation

Simple emulsion (O/W) is an effective technique for encapsulating hydrophobic molecules. In this method, the polymer and the drug are dissolved in volatile organic solvents such as dichloromethane, chloroform, or ethyl acetate. This solution is then emulsified in an aqueous phase containing a surfactant, such as poly­(vinyl alcohol) (PVA), under continuous mechanical agitation. The volatilization or extraction of the organic solvent leads to the formation of nanoparticles.^76,97^

#### 4.1.2 Double Emulsion-Solvent Evaporation

The double emulsion-solvent evaporation (W/O/W) method is employed to encapsulate hydrophilic drug molecules, addressing the limited encapsulation efficiency (EE) often observed with the single emulsification technique. In this approach, an aqueous solution containing the drug is first emulsified in an organic phase comprising PLGA under vigorous agitation, forming W/O droplets. This emulsion is then introduced into an external aqueous solution containing a surfactant, resulting in the formation of W/O/W droplets. The subsequent removal of the organic solvent through evaporation allows the production of nanoparticles.^75,76,98^

Despite their widespread use and effectiveness, nanoparticles prepared via this method may exhibit low EE due to the diffusion of hydrophilic drugs into the external aqueous phase during nanoparticle formation.^99^

Both techniques are ideal for laboratory-scale synthesis, but on an industrial scale, they are limited to lipophilic drugs and demand substantial energy input during homogenization. Nonetheless, adjustments in process parameters, such as stirring speed and temperature, have been shown to effectively mitigate some limitations associated with these methods.^73^

### 4.2 Salting Out

In this method, a solution comprising PLGA and the drug, dissolved in a water-miscible organic solvent, is introduced into an aqueous phase containing a salting agent, such as calcium chloride, along with a stabilizer like PVA. Continuous stirring leads to the initial formation of an oil-in-water (O/W) emulsion. Following this, a large volume of water is added, facilitating the diffusion of the organic solvent into the aqueous phase, which results in nanoparticle formation. Finally, the salting-out agents are removed through filtration, and the nanoparticles are thoroughly washed to eliminate excess stabilizer.^78,82,100^

The primary benefit of the salting-out technique lies in its ability to minimize stress on protein encapsulants and since salting out does not require elevated temperatures, it is especially advantageous for processing heat-sensitive substances. However, significant drawbacks include its limitation to lipophilic drugs and the need for extensive washing steps to remove residual salts and solvents.^100^

### 4.3 Solvent Emulsification-Diffusion (ESD)

The method proposed by Quintanar-Guerrero et al.^101^ is a modified version of the salting-out technique that employs nanoprecipitation for nanoparticle formation. Initially, the organic solvent and water are mutually saturated at room temperature to establish a thermodynamic equilibrium. Subsequently, a solution containing both the polymer and the drug is prepared in the organic solvent and emulsified in an aqueous solution containing a surfactant, typically PVA, using a high-speed homogenizer. Following emulsification, water is gradually added to the oil-in-water (O/W) emulsion under constant agitation, facilitating phase transformation and the external diffusion of the solvent. This process results in the formation of colloidal nanoparticles through the nanoprecipitation of the polymer. Finally, the solvent is removed via vacuum distillation or evaporation.^73,75^

This method presents several advantages, including high EE, reproducibility between batches, ease of scale-up, and simplicity of execution. Additionally, it results in a narrow particle size distribution, enhancing the uniformity of the final product. However, it is not without its drawbacks. The need to remove large volumes of water from the suspension can complicate the process, and there is a risk of water-soluble drugs leaking into the external aqueous phase during nanoparticle formation, which may affect the overall encapsulation efficiency.^102^

### 4.4 Nanoprecipitation

The nanoprecipitation method represents a straightforward and effective technique primarily employed for the encapsulation of hydrophobic drugs.^75,78^ This single-step process, commonly referred to as the solvent displacement method,^73,95,100^ initiates with the dissolution of both the polymer and the drug in a water-miscible organic solvent, such as acetonitrile, ethanol, methanol, or acetone. Subsequently, the organic solution is introduced dropwise into an aqueous solution that contains a surfactant. The rapid diffusion of the organic solvent into the aqueous phase promotes the immediate formation of PLGA nanoparticles.^79,100^

Although the nanoprecipitation method is predominantly employed for encapsulating hydrophobic drugs, several modifications have been introduced to facilitate the encapsulation of hydrophilic molecules. One notable approach involves substituting water with alternative liquids, such as cottonseed oil and Tween-80, which create a nonsolvent phase that enhances the precipitation of PLGA.^75^ Additionally, a variant of the two-step nanoprecipitation method has been widely adopted for protein encapsulation. In the initial stage, the protein is precipitated, followed by a second stage where the precipitated protein is encapsulated by PLGA, resulting in nanoparticles with high EE.^103^

This method offers advantages such as scalability, low energy requirements and good reproducibility. The properties of the resulting nanoparticles are influenced by factors including polymer content and molecular weight, the characteristics of the solvents used, and the solvent-to-polymer ratios, along with the mixing rate applied during synthesis.^78^ The disadvantages associated with nanoprecipitation are also inherent to other methods, including the removal of potentially toxic impurities, such as residual organic solvents, excess surfactants, unreacted monomers, and large polymer aggregates.^95^

### 4.5 Dialysis

The dialysis technique is frequently employed for the production of small nanoparticles. In this method, the polymer is initially dissolved in a volatile organic solvent and then placed within a dialysis bag featuring appropriately sized pores. The dialysis membrane facilitates the separation of the solvent and antisolvent. As the solvent gradually diffuses out of the bag, the solubility of PLGA diminishes. This process leads to the progressive aggregation of the polymer, ultimately resulting in the formation of a homogeneous suspension of nanoparticles.^73,79^

This approach offers a simple, cost-effective setup, reduced energy consumption, and moderate conditions suitable for processing sensitive drugs. However, this method is limited to small batch preparations, as scaling up production may lead to changes in the properties of the nanoparticles.^79^

The previously mentioned approaches result in the production of NP suspensions that can be administered by nebulizers. This route device is widely recognized for its simplicity in terms of industrial viability and its lower impact on the physicochemical properties of NPs. However, traditional nebulizers have significant limitations, such as high losses of nebulized material to the environment, which compromises precise control of the administered dose.^51^

In this context, it is necessary to subject the NP produced to drying processes, such as freeze-drying and spray-drying. However, these techniques present significant challenges, including the precise control of the diameter of the NPs, the scaling up to adequate processing volumes, and the need to use carriers.^51^ The freeze-drying process can result in the formation of irreversible aggregates, which renders this approach unsuitable for the drying of nanoformulations. Conversely, spray-drying enables the nanoformulations to be dried rapidly and with greater control over particle size. However, this technique is costly and is only applicable to nonthermosensitive drugs.^51,64^ Consequently, the synthesis of nanoparticles directly in the solid state has emerged as a promising alternative to overcome these limitations.

### 4.6 Spray-Drying

The spray-drying technique is extensively utilized for the preparation of polymer-based nanoparticles owing to its practicality and scalability in industrial applications.^72^ In this process, solutions, suspensions, or emulsions containing polymers and drugs are atomized through a nozzle and injected into a stream of hot air. The rapid evaporation of the solvent leads to the formation of solid particles. This method comprises four primary stages: atomization, mixing of droplets with dry gas, volatilization of the solvent, and separation of the product.^76^ Critical parameters that influence particle size distribution include the selection of the atomizer, nozzle pressure, and feed rate.^72^

Spray-drying offers numerous advantages, including the capability to generate particles rapidly and continuously in a single step, thereby eliminating the necessity for additional drying processes.^104^ The automated nature of this process can yield high encapsulation efficiencies, rendering it suitable for encapsulating a diverse array of molecules, such as proteins, peptides, plasmid DNA, and small molecules.^104,105^ This technique is effective for both hydrophilic and hydrophobic substances, as it does not necessitate an external solvent phase.^72,76^ However, challenges such as the adhesion of nanoparticles to the inner walls of the dryer can hinder efficient particle collection. This issue can be alleviated by appropriately optimizing the processing variables.^72^

Compared to other preparation techniques, spray-drying is particularly notable for its ability to yield solid formulations directly, thereby eliminating the necessity for additional drying steps. This characteristic enhances the speed and efficiency of the process, making it especially advantageous for the production of PLGA nanoparticles intended for pulmonary administration.^106^

## 5 PLGA-Based Nanoparticles for Pulmonary Drug Delivery

PLGA NPs have been employed for the direct delivery of drugs to the lungs in the treatment of various pulmonary diseases. This approach not only enhances therapeutic efficacy but also minimizes systemic toxicity and improves patient adherence to treatment regimens.^76^ Lung administration offers several advantages, including the avoidance of the hepatic first-pass effect, as well as leveraging the lung’s extensive absorption surface area, thin epithelial membrane, and robust blood supply.^109–111^ To facilitate effective targeting of the lungs, NPs can be formulated as inhalable dry powder preparations, necessitating the optimization of their aerodynamic diameters to ensure deeper retention within the pulmonary system.^76,109^

Given that lung mucus is rich in negatively charged groups, PLGA NPs functionalized with positively charged molecules can enhance mucoadhesive properties, thereby improving the efficacy of the delivery system. Mucoadhesion represents a valuable strategy for optimizing pulmonary drug delivery, as it prolongs the retention time of the drug within the lung, ultimately enhancing therapeutic outcomes.^20,111^

Cellular uptake of NPs is markedly influenced by their surface charge. In the study by Areny-Balagueró et al.^136^ PLGA NPs were synthesized for pulmonary delivery via intratracheal instillation. The NPs, labeled with a red fluorescent dye, were engineered to exhibit either a positive (Cy5/PLGA^+^) or negative (Cy5/PLGA^–^) surface charge. Notably, Cy5/PLGA^–^ NPs demonstrated faster and more extensive cellular internalization in both macrophages (THP-1) and alveolar epithelial cells (HPAEpiC) compared to their positively charged counterparts. Moreover, in vivo studies revealed that Cy5/PLGA^–^ NPs were retained in all lung lobes 1 h postinstillation and preferentially accumulated in pulmonary macrophages after 24 h. These results highlight the potential of negatively charged PLGA nanoparticles as an effective platform to enhance pulmonary cellular uptake in the evaluated models.

Due to their distinctive properties and numerous advantages, extensive research has focused on the pulmonary administration of drugs encapsulated in PLGA-based nanoparticles, yielding highly promising outcomes. Although several studies have explored the systemic delivery of PLGA nanoparticles, as reviewed by Cai et al.^112^ in the context of the nervous system, by Lin et al.^113^ in the administration of sorafenib for the treatment of liver fibrosis, and by Liu et al.^114^ in the delivery of insulin via the lungs, the present study focuses on the treatment of lung diseases and clinical syndromes with an emphasis on local delivery. A summary of the key findings from these studies is presented in Table 1.

**Table 1:** PLGA-Based Nanoparticles for Pulmonary Drug Delivery^a^

disease	drug	limitations	carrier	main results	refs
asthma	glycyrrhizic acid	low efficacy and reduced adverse effects on airway	PLGA NPS	decreases the inflammatory response in the airways and vessels; reduces mucus hypersecretion and airway obstruction, preventing shortness of breath	^7^
pulmonary fibrosis	simvastatin (SV)	low water solubility and chemical instability	PLGA NPS	reduced progression of pulmonary fibrosis in vivo by decreasing anti-inflammatory markers, particularly at the 10 mg SV dose; NP size parameter was found to be effective for alveolar position and mucus penetration, allowing NPs to diffuse through mucus	^8^
	tacrolimus	low water solubility and narrow therapeutic index	CS-coated PLGA NPs	direct inhalation twice weekly showed superior antifibrotic efficacy compared to daily oral tacrolimus in mice with bleomycin-induced pulmonary fibrosis, reducing the frequency of administration and associated side effects	^9^
cystic fibrosis	ciprofloxacin	pH dependent solubility and limited organic solvent solubility	PLGA NPS	enhanced antimicrobial activity against in vitro and showed no toxicity against bronchial epithelial cell lines in vitro	^10^
lung cancer	gemcitabine hydrochloride (GEM) and paclitaxel (PTX)	limited therapeutic efficacy, severe off-target toxicity effects and poor long-term survival	gemcitabine-loaded PLGA-NPs coated with lung surfactant (infasurf) containing paclitaxel	increased the retention time of the anticancer drug gemcitabine in lung tissues; decreased the absorption of nanoparticles by alveolar macrophages in vitro; decreased cancer cell survival and colony formation in vitro	^6^
	silibinin (SB)	high administration frequency and systemic toxicity	CS coated PLGA NPs	CS coated PLGA NPs induced cell inhibition activity against A549 cell line; deep lung deposition and cellular adhesion; increased the rate and extent of SB bioavailability in vivo	^11^
tuberculosis	*N*-acetylcysteine	low bioavailability which affects the drug therapeutic efficacy	PLGA NPS	increased antibacterial activity against in vitro (MTB H37Rv); in vitro lung deposition study showed favorable results in terms of deposition of the emitted dose and targeting to the lungs and sustained release (up to 48 h) with greater retention for tuberculosis management	^12^
	linezolid	low solubility in water, short plasma half-life and unwanted side effects	PLGA NPS	improved deep lung deposition in vitro	^13^
	ethionamide	short half-life and small fraction reaching lungs	PLGA NPs	controlled release in vitro; improved deep lung deposition in vivo	^14^
acute respiratory distress syndrome (ARDS)	human serum albumin (HSA)	low lung drug delivery efficiency	PLGA NPs	PLGA/HAS NPs showed good postnebulization stability and broad pulmonary biodistribution	^135^

a ARDS: Acute respiratory distress syndrome; ATRA: All-trans retinoic acid; CS: chitosan; GEM: Gemcitabine hydrochloride; HAS: Human serum albumin; PLGA NPs: poly­(lactic-*co*-glycolic) nanoparticles; PTX: paclitaxel; SB: Silibilin; SV: Simvastatin.

### 5.1 Asthma

Asthma is a multifaceted disease characterized by irreversible airway obstruction, hyperresponsiveness, and chronic inflammation, leading to airway wall remodeling.^116^ Current therapeutic strategies primarily target symptom relief through the use of bronchodilators, β-2 agonists, and glucocorticosteroids. While asthma attacks are effectively managed with glucocorticoids and long-acting β-2 agonists,^117^ reliance on high doses of these medications has been associated with diminished clinical efficacy and potential harm. Furthermore, inadequately controlled inflammation may precipitate sudden death in severe asthma cases. Thus, there is an urgent need to develop alternative therapeutic strategies that can effectively address airway remodeling and hypersensitivity, minimizing the risk of severe adverse effects.^38,118^

Chen et al.^7^ investigated the therapeutic potential of PLGA nanoparticles (NPs) encapsulating glycyrrhizic acid for the treatment of allergic asthma in an in vivo model. The NPs exhibited an average diameter of 350 ± 50 nm, and a drug release of 67% was observed after 10 h. The ζ-potential demonstrated an efficient encapsulation of the drug. Following the administration of the aerosolized NP solution, significant reductions in interleukin-5 (IL-5) and interleukin-13 (IL-13) levels were noted in the bronchoalveolar lavage fluids of the BALB/c mouse group compared to the untreated control group. Additionally, histological analysis revealed a decrease in mucus hypersecretion in the airways and cell hyperplasia in the bronchi of the NP-treated group relative to both the untreated group and the group receiving budesonide.

Athari et al.^119^ formulated PLGA nanoparticles (NPs) encapsulating vasoactive intestinal peptide (VIP), a compound recognized for its antispasmodic and anti-inflammatory properties, positioning it as a promising alternative for asthma management. The resulting PLGA-VIP nanoparticles demonstrated an average diameter of 550 ± 50 nm. A noticeable shift in the ζ-potential toward a more positive charge confirmed the successful encapsulation of positively charged peptides within the PLGA matrix. The NPs were subsequently freeze-dried to produce a dry powder formulation. These NPs demonstrated a sustained release profile, with 78% of VIP being released over a 10 h period at a pH representative of the bronchoalveolar environment. Given their average diameter, these NPs are anticipated to be suitable for deposition within the deeper regions of the lungs, enhancing their therapeutic potential.

### 5.2 Pulmonary Fibrosis

Pulmonary fibrosis (PF) is a chronic pulmonary disorder characterized by the progressive loss of lung epithelial cells coupled with the accumulation of fibroblasts,^120^ which in turn stimulates collagen synthesis. This pathological process severely impairs respiratory function and gas exchange capabilities. Patients frequently encounter sudden declines in respiratory capacity,^121^ and the mortality rate associated with PF exceeds 70%, highlighting the critical need for effective therapeutic interventions.^9,122^

Shahabadi et al.^8^ encapsulated simvastatin (SV) in PLGA NPs (PLGA-SV NP) as an alternative for the treatment of PF in rats with paraquat (PQ)-induced lung injury. The NPs had an average diameter of 222.5 ± 5.245 nm, with a negative ζ-potential. In addition, the encapsulation efficiency of SV in PLGA NPs was approximately 62.3%. The release profile was controlled, with 51% of SV released in 16 h. Groups of rats with induced lung injury were treated with the developed NPs via nebulization, and in vivo tests showed that the levels of the cytokines IL-6 and TNF-α in the blood serum of the groups treated with inhalable PLGA-SV NP (10 mg/kg/day) decreased significantly (Figure 5). The treatment also almost completely reduced the pathological changes in the lungs and the ability to contractile response of the trachea to methacholine.

**Figure 5:**
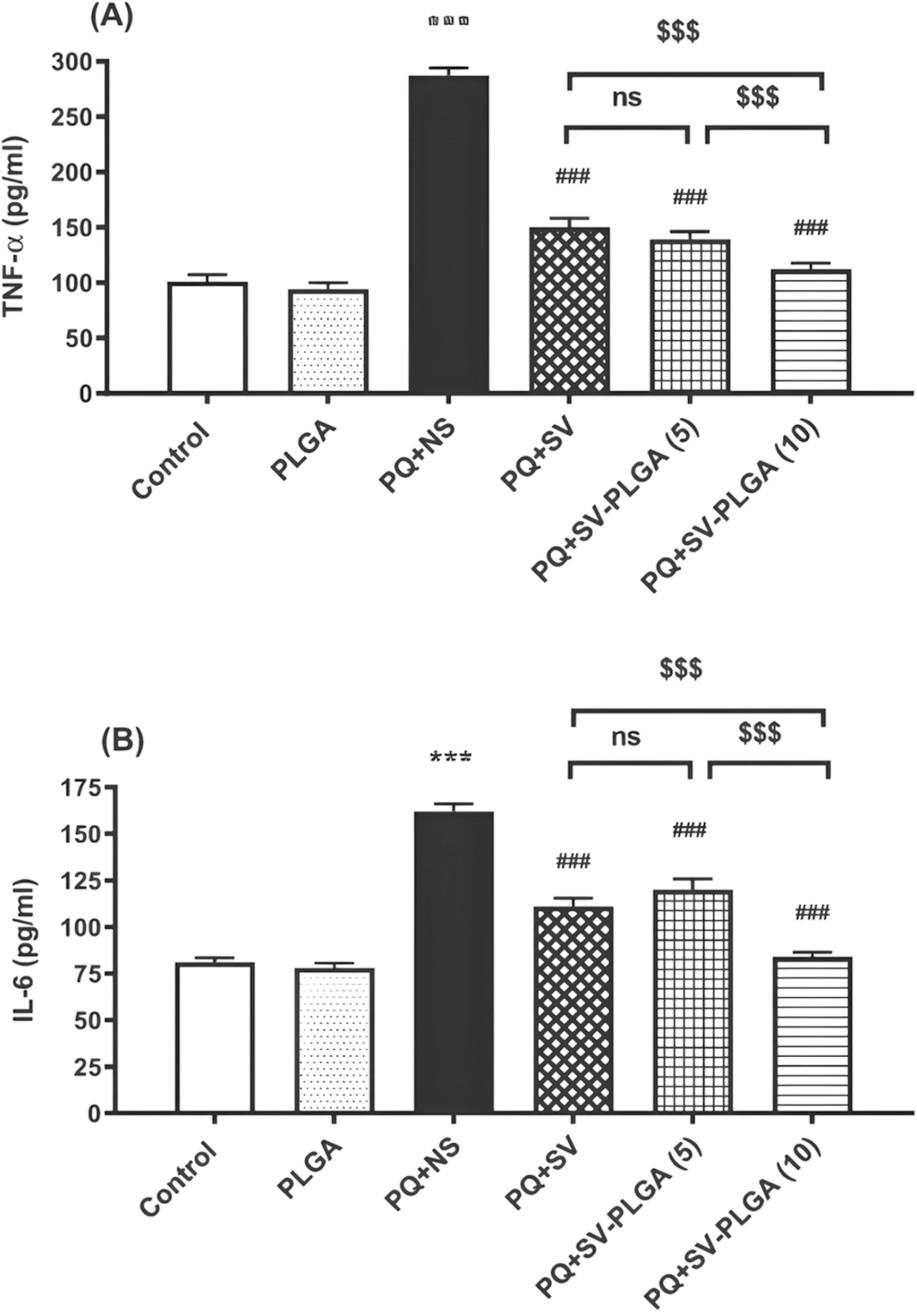
The comparison of TNF-α (A) and IL-6 levels (B) was conducted among the control group, a group exposed to an aerosol of paraquat (PQ) at 54 mg/m^3^, and groups treated with PQ combined with 5, 10, or 20 mg/kg/day of PLGA-SV nanoparticles (PLGA-SV NP) or PQ plus simvastatin (SV) at 20 mg/kg. Statistical significance was observed with ***p < 0.001 compared to the control group, ###*p* < 0.001 compared to the PQ group, and $$$ indicating significant differences between treatment groups. The data are presented as mean ± SEM, with *n* = 6 for each group. Reproduced with permission from ref 8. Copyright 2022 John Wiley and Sons.

In the study by Lee et al.^9^ CS-coated PLGA NPs were developed for the encapsulation of tacrolimus (TAC) (CS-TAC PLGA NPs). Due to its mucoadhesive characteristics, CS was employed to coat the NPs, thereby enhancing the pulmonary retention and absorption of TAC in the lungs. The NPs were aerosolized using a microspray for pulmonary administration in C57BL/7 mice with bleomycin-induced pulmonary fibrosis. The NPs had an average diameter of 441 ± 11.9 nm and the ζ-potential measurements revealed values of −28.3 ± 1.1 mV for TAC PLGA NPs and +13.6 ± 0.9 mV for CS-TAC PLGA NPs, highlighting the charge reversal upon CS coating. CS-TAC PLGA NPs exhibited a controlled release profile without significant burst release, with the drug being gradually released over approximately 5 days.

In vivo studies of lung histology revealed that there was a significant reduction in fibrosis in the group treated with CS-TAC PLGA NPs twice a week when compared to the control group and the group treated with TAC orally daily.

### 5.3 Cystic Fibrosis

Cystic fibrosis (CF) is caused by dysfunction of mucociliary clearance from the airways, due to a mutation in the cystic fibrosis transmembrane conductance regulator (CFTR) gene.^123^ This mutation results in impaired mucociliary clearance, leading to mucus accumulation, increased susceptibility to bacterial infections, exacerbated inflammatory response and airway obstruction. There are various treatments for CF, including antibiotics, bronchodilators, pancreatic enzyme replacement, respiratory physiotherapy, mucolitics and steroids.^123,124^

In this context, Türeli et al.^10^ evaluated PLGA NPs containing ciprofloxacin as a proposal for the treatment of CF. The NPs developed had an average diameter of 190.4 ± 28.6 nm and a ζ-potential of −22.5 ± 5.4 mV. Encapsulation efficacy of 79.3 ± 0.9% was achieved. The in vitro release behavior of the nanoparticles was assessed in three media relevant to in vivo conditions: phosphate-buffered saline (PBS) at pH 7.4 with or without 0.2% Tween-80 (T80), and simulated lung fluid (SLF). The results demonstrated a controlled release profile of ciprofloxacin over 8 h, with no significant burst release observed during the initial sampling period. In water, ciprofloxacin release reached 59.2% ± 3.0% after 8 h. In contrast, in all in vivo-relevant media, approximately 80% of the drug was released within 8 h, followed by a very gradual release over the subsequent 14 days, achieving a cumulative release of approximately 90.5%. In vitro turbidimetric measurement tests of the interaction of horse lung mucus with NPs show that PLGA NPs containing the drug exhibited size and surface properties that facilitated penetration into the thick, negatively charged mucus present in CF lungs, where bacteria reside. The controlled release of the antibiotic over 8 h is expected to maintain a high and sustained local concentration of the drug. In addition, the enhanced antibacterial activity of nanostructured ciprofloxacin against suggests that a reduced dose can be administered, thus minimizing adverse effects. In addition, the in vitro bioluminescence cytotoxicity assay (ToxiLight) against Calu-3 HTB-55 and CFBE41o-strains showed that the NPs developed were safe for healthy bronchial epithelial and CF cells, even at concentrations higher than those reported for ciprofloxacin in vivo in the lung.

Al-Nemrawi et al.^125^ developed CS-coated PLGA NPs for the encapsulation of Tobramycin (TB), an antibiotic class drug widely used to treat lung infections caused by . Coating with CS would increase the efficacy of the NPs by increasing their residence time in the lung mucus, a consequence of their mucoadhesive properties. The nanoparticles developed had an average diameter in the range of 309.57 ± 1.12 to 575.77 ± 2.67 nm and a ζ-potential between +33.47 ± 1.0 mV and +50.1 ± 6.5. The encapsulation efficiency of TB in the NPs ranged from 83.74% to 88.47%. The in vitro release profile was performed and pure drug exhibited rapid dissolution, with 99% availability in solution within 30 min. All formulations demonstrated an initial burst release within the first 2 h, followed by a more gradual release phase. Notably, tobramycin release from coated nanoparticles was slower compared to uncoated ones. Over 2 days, uncoated PLGA nanoparticles released 86.82% ± 2.3% of the encapsulated drug. In contrast, the coated nanoparticles exhibited reduced release rates of 71.81% ± 3.1%, 65.52% ± 1.8%, and 59.53% ± 2.0% for formulations for the formulations tested.

The mucoadhesiveness test was evaluated by measuring the change in ζ-potential as the NPs interacted with mucin, which is negatively charged. The results showed that the values decreased slightly over the incubation time, revealing that there is interaction with the mucus. The MIC test was carried out against strains of (PA01). The nanoparticles were compared to the free drug and to nanoparticles without drug. All the formulations, with the exception of the TB-free NPs, were able to inhibit bacterial growth. A close relationship was also observed between the concentration of CS used in the formulation and bacterial growth, since higher quantities of CS showed lower MIC values, proving the antimicrobial property of this polymer.

### 5.4 Lung Cancer

Lung cancer has the highest mortality rate of all common cancers in men and the second in women,^126^ with a survival rate of less than five years. Currently, although chemotherapy remains the mainstay of treatment for advanced lung cancer, most traditional chemotherapy drugs have significant limitations, including poor selectivity, low bioavailability and serious side effects.^127,128^

In the study conducted by Raval et al.^11^ an inhalation powder formulation containing silibinin (SB) encapsulated in PLGA NPs coated with CS was developed. The optimized formulation exhibited a diameter of 284 ± 0.47 nm, a ζ-potential of 22.5 ± 0.78 mV, and an encapsulation efficiency of 56.8 ± 0.87%. Moreover, the in vitro release profile indicated that 72.5% of SB was released from the nanoparticles after 48 h. The MTT test carried out for the in vitro toxicity study showed that the NPs showed high cell inhibition activity against the A549 strain when compared to the control group (free-drug NP), revealing their anticancer activity. The lyophilized NPs had a fine particle fraction of 80.2%, demonstrating their efficacy in pulmonary delivery. After administration of the formulation via modified DPI, an in vivo biodistribution study showed that these nanoparticles penetrated the deepest layers of lung tissue, providing both local and systemic actions of the drug, promoting cell adhesion and pulmonary retention of the NPs in Sprague–Dawley rats. The histopathology study on lung epithelial tissue to assess in vivo toxicity demonstrated the safety of the formulation to be administered to living organisms, since no tissue damage was observed in the lungs of rats treated with the inhalation formulation. The in vivo pharmacokinetic study in rats indicated a significant improvement in the rate and extent of SB bioavailability from CS-coated PLGA NPs. Therefore, CS coating on PLGA NPs showed promise for increasing bioavailability in pulmonary administration and may be useful in the treatment of lung cancer.

In the research conducted by Elbatanony et al.^129^ inhalable PLGA NPs containing Afatinib (AFA-NP) were developed to improve the therapeutic response in patients with nonsmall cell lung cancer (NSCLC). Physicochemical characterization showed a mean diameter of 180.2 ± 15.6 nm and a ζ-potential of −23.1 ± 0.2 mV for the AFA-NPs obtained. An encapsulation efficiency of 34.4 ± 2.3% was obtained. The in vitro release assay in phosphate-buffered saline, pH 7.4 at 37 °C revealed that 56.8 ± 6.4% of the FFA had been released within 48 h, indicating a prolonged release profile. In vitro pulmonary deposition tests of the dry powder were carried out using the Next-generation cascade impactor. The fine particle fraction (FPF) of 77.8 ± 4.3% demonstrates good aerosolization capacity, and the Mass Median Aerodynamic Diameter (MMAD) of 4.3 ± 0.2 μm indicates that AFA-NPs can reach the deeper layers of the lungs. In vitro cytotoxicity studies were carried out on two human cancer lines, A549 and H460 using the MTT assay. The results revealed that AFA-NPs considerably increased the cytotoxic potential of AFA in the aforementioned cell lines. To mimic a lung tumor, 3D spheroids with A549 cells were cultured and treated with control, AFA-NP and free AFA to predict the physiological interaction of the tumor mass with the developed nanoparticles. The study was conducted as a single or multiple treatment, and for each treatment, concentrations of 1 and 3 μM were administered. Images were taken (Figure 6) and the results observed show that AFA-NPs have greater efficacy in penetrating the tumor and inhibiting its growth, when compared to free AFA and control.

**Figure 6:**
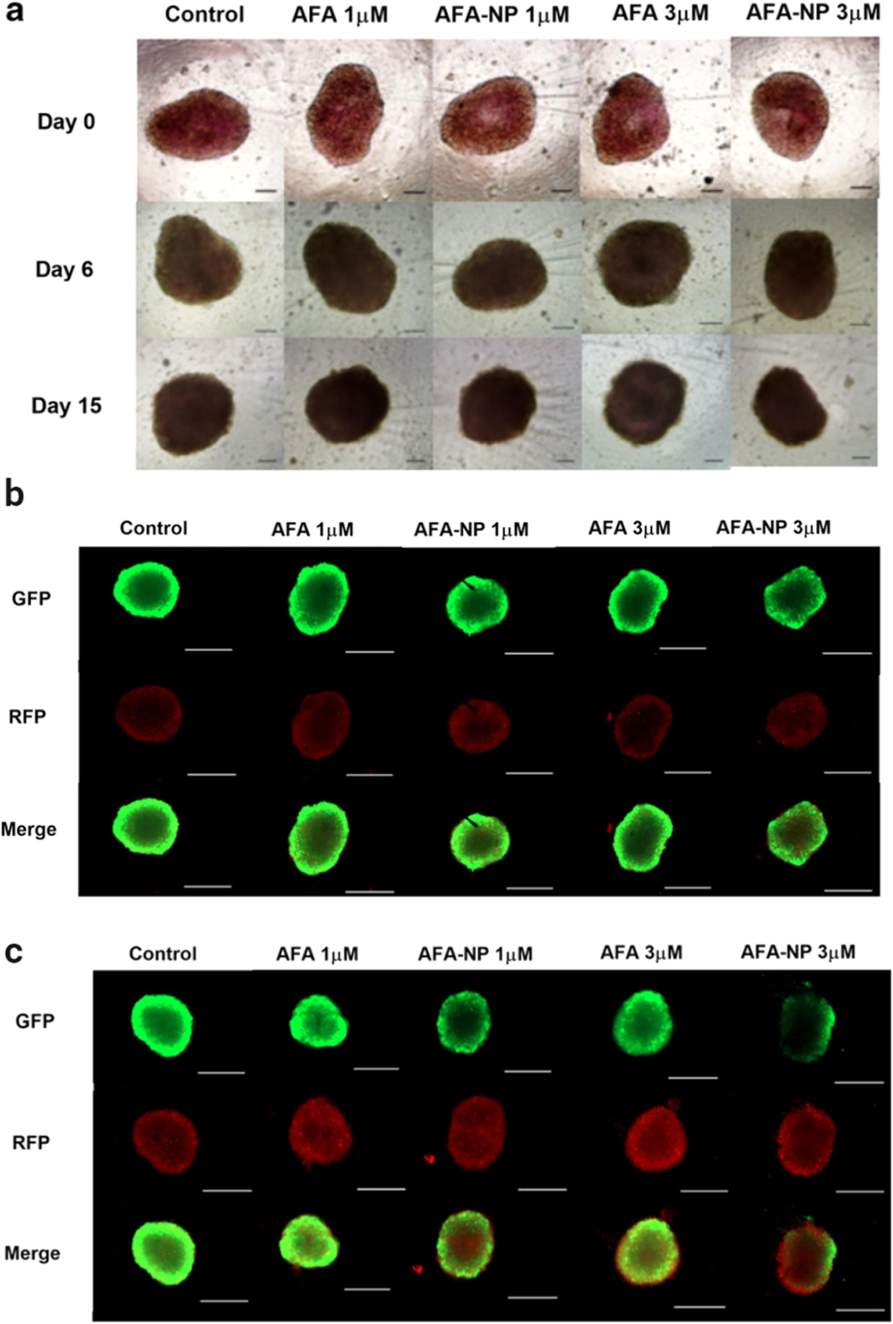
3D spheroid study: single dosing regimen: A549 cells were treated with control, free AFA and AFA-NP (1, 3 μM). (a) Images represent spheroids at 0, 6, and 15 days of treatment. (b) Green (GFP) regions indicate live stained cells and red (RFP) dead stained cells. The merged images indicate the overlap of GFP and RFP regions. Based on the results, it can be stated that AFA-NP demonstrated a significant reduction (*p* < 0.05) in tumor progression after a single treatment dose, administered for 6 and 15 days, at concentrations of 1 and 3 μM, compared to treatment with free AFA. (c) In multiple-dose treatment, AFA-NPs showed a significant reduction (*p* < 0.05) in tumor progression, especially after 6 days of treatment at concentrations of 1 and 3 μM in the A549 NSCLC cell line compared to treatment with the free drug. Adapted image reproduced with permission from ref 129. Copyright 2020 Springer Nature.

### 5.5 Tuberculosis

Tuberculosis (TB) is a lung infection caused by (Mtb). Available treatment includes chemotherapy with first-line drugs, administered orally: isoniazid, pyrazinamide, rifampicin and ethambutol. In addition, second-line drugs such as streptomycin, amikacin, kanamycin, viomycin and capreomycin, administered parenterally, and other oral drugs can also be used. The WHO recommends daily oral administration of isoniazid, pyrazinamide, rifampicin and ethambutol for 2 months, followed by isoniazid and rifampicin for a further 4 months. However, if the first-line drugs fail, it is necessary to resort to second-line drugs, which are more toxic and expensive. Even the new molecules, such as bedaquiline and delamanid, are toxic. Therefore, the need for high daily doses for long periods and the toxic effects associated with the drugs make adherence to treatment and curing the disease a significant challenge. In addition, many strains of Mtb are multiresistant to antimicrobial agents. These disadvantages justify the administration of these molecules directly into the lungs for the local treatment of TB, with a focus on reducing the dose and side effects.^130^

*N*-Acetylcysteine (NAC) has synergistic, hepatoprotective and otoprotective effects in relation to anti-TB drugs, as well as acting as an antioxidant, anti-inflammatory, mucolytic and antimycobacterial agent by increasing the production of interleukins and interferon-γ (INF-γ). In this sense, the study conducted by Puri et al.^12^ sought to develop PLGA NPs coated with Pluronic F127 containing NAC (NAC-PLGA-MPPs) for inhalation administration, with a view to their antimycobacterial action. The optimized NAC-PLGA-MPPs had an average diameter of 382.63 ± 6.42 nm, a ζ-potential of −14.3 ± 2.1 mV and encapsulation efficiency of 55.46 ± 2.40%. The release profile of NAC-PLGA-MPPs demonstrated a biphasic pattern, beginning with an initial burst release of 64.67 ± 1.53% within the first 12 h, followed by a sustained release phase reaching approximately 76.33 ± 0.57% over 48 h. The nanoparticles were freeze-dried to obtain a dry powder. The in vitro deposition tests carried out with the powder for inhalation on the Next-generation Cascade Impactor showed that the particles can be deposited in the deeper layers of the lungs and have a size in the range of 1–5 μm, reaching the alveolar region. In addition, the flow properties of the powder were determined from the angle of repose of the powder and the Hausner ratio, and the results indicated good fluidity of the powder containing NAC-PLGA-MPPs, showing that PLGA nanoparticles constitute an ideal delivery system for penetration into lung mucus. Finally, NAC-PLGA-MPPs showed greater antimycobacterial activity in vitro when compared to free NAC against the H37Rv strain of Mtb, showing that NAC-PLGA-MPPs can be an effective adjuvant therapy in the treatment of TB.

In the same context, Shah et al.^13^ used linezolid (LZ) to be encapsulated in PLGA NPs (LZ NPs) for the development of an inhalation formulation. The optimized formulation exhibited a mean particle size of 45.2 nm, an encapsulation efficiency of 85.33%, and a drug release of 89.84% over 120 h. In vitro deposition studies using Anderson Cascade Impactor ensured that the inhalation powder developed containing LZ NPs had the appropriate aerodynamic characteristics to reach the deepest layers of the lungs for the effective treatment of TB (MMAD of 3.78 μm). In addition, the Minimum Inhibitory Concentration (MIC) study against Mtb revealed that the LZ NPs developed had lower MICs and were more effective at inhibiting Mtb growth when compared to the free drug.

PLGA NPs containing ethionamide (ETH), were developed and converted into dry inhalation powder (DPI) through lyophilization by Debnath et al.^14^ The optimized formulation demonstrated a particle size of 488.4 ± 7.2 nm, a ζ-potential of −5.7 ± 0.3 mV, and an encapsulation efficiency of 83.62 ± 0.97%. Moreover, the in vitro release profile of the lyophilized powder revealed an initial burst release of 9.23 ± 1.15% within the first hour, followed by a controlled release of 95.17 ± 3.59% over 24 h. The aerodynamic properties were evaluated using an 8-stage cascade impactor, and the DPI showed adequate MMAD results for pulmonary administration (1.79 μm). In addition, the lyophilized powder showed good fluidity, as revealed by the Carr Index, Hausner ratio and angle of repose. In vivo studies were carried out to evaluate the biodistribution of ETH NP and free ETH in the lungs and plasma. The results showed that the prepared DPI maintained the concentration of ETH above the minimum inhibitory concentration (MIC) for more than 12 h after administration of a single dose in rats. Thus, the authors argued that the product developed can improve treatment efficacy by raising the concentration of ETH in lung tissue with a single, reduced dose.

### 5.6 Acute Respiratory Distress Syndrome (ARDS)

Acute respiratory distress syndrome (ARDS) is a clinical syndrome characterized by acute hypoxemic respiratory failure secondary to a severe inflammatory insult in the lungs. It encompasses a heterogeneous group of etiologies that converge on common clinical and pathophysiological hallmarks, including increased alveolar-capillary membrane permeability leading to inflammatory edema, accumulation of nonaerated lung tissue with reduced compliance (increased elastance), and significant venous admixture and dead space ventilation, ultimately resulting in hypoxemia and hypercapnia.^131,132^ Over the years, multiple therapeutic strategies have been investigated for ARDS, aiming to address its underlying pathophysiological mechanisms at various stages of the disease. Experimental treatments have focused on mitigating alveolar epithelial injury, modulating inflammatory and immune responses, controlling edema and fibrosis, and limiting vascular remodeling, endothelial permeability, and cellular damage.^133^

In this context, Solé-Porta et al.^135^ developed PLGA nanocapsules encapsulating human serum albumin (PLGA/HSA NCs) for pulmonary delivery via nebulization, targeting complex pulmonary pathologies such as ARDS. The PLGA/HSA NCs exhibited a mean particle size ranging from 200 to 210 nm before and after nebulization in water. When nebulized in saline, the mean particle size increased from 190 nm prenebulization to 230 nm postnebulization. Aerosols generated with water as the dispersion medium produced droplets approximately 6 μm in diameter, whereas those generated with saline yielded droplets around 3 μm, closer to the optimal aerodynamic size for efficient pulmonary deposition. In vivo biodistribution studies, conducted using a vibrating mesh nebulizer, evaluated pulmonary retention in healthy and acute lung injury (ALI) Sprague–Dawley rats following administration of Cy5-labeled PLGA/HSA NCs (PLGA-Cy5/HSA NCs). Fluorescence molecular imaging confirmed the presence of PLGA-Cy5/HSA NCs in the lungs of both healthy and ALI animals 16 h postnebulization, as evidenced by increased fluorescence intensity relative to untreated controls (Figure 7). Cellular uptake of the nanocapsules by alveolar type II (ATII) cells was significantly higher in healthy animals compared to ALI-induced rats, likely attributable to increased alveolar-capillary membrane permeability in the injured lungs. Moreover, nebulization appeared to reduce macrophage-mediated pulmonary clearance of the PLGA NCs, thereby enhancing their tissue retention. Collectively, these findings underscore the potential of PLGA NPs as promising carriers for noninvasive pulmonary drug delivery, given their ability to maintain physicochemical integrity postnebulization, generate aerosols with suitable aerodynamic properties, achieve widespread lung distribution, and selectively target ATII cells—critical mediators of lung immunity and repair.

**Figure 7:**
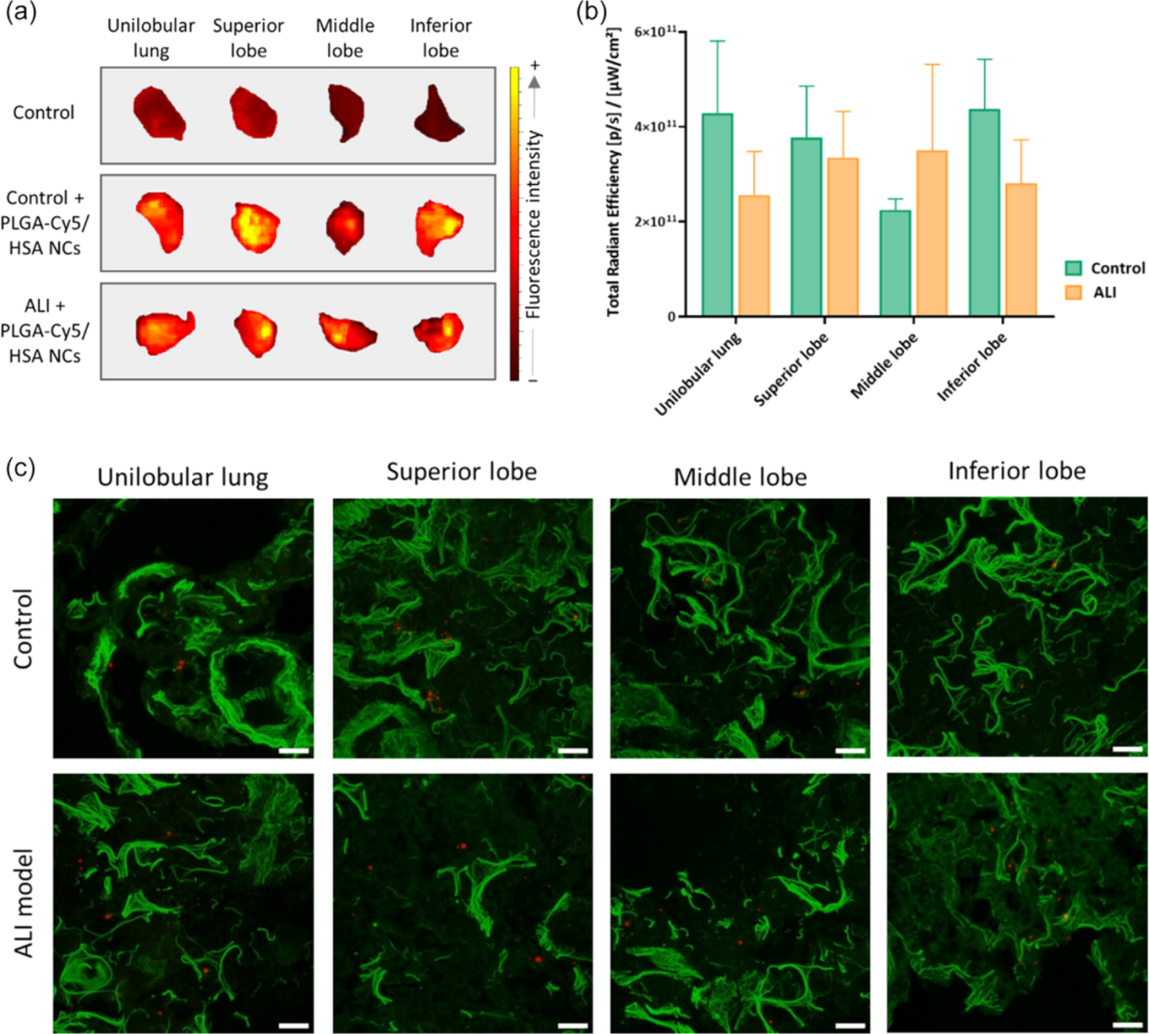
In vivo biodistribution of PLGA-Cy5/HSA NCs in healthy and ALI animals: (a) FMI views of the lungs of a healthy animal nebulized with NCs (control), and an ALI animal nebulized with NCs, confirming the pulmonary retention of PLGA-Cy5/HSA NCs in both groups; (b) corrected total radiant efficiency (TRE) on unilobular lung, superior, middle, and inferior lobes regions of interest (ROIs) of control and ALI animals nebulized with NCs. Although TRE in the lungs of ALI animals was lower than in healthy counterparts, the difference in NC retention between healthy and injured lungs was not statistically significant and; (c) *Z*-stacking analysis of lung (unilobular lung, superior, middle, and inferior lobes) tissue slices of animals nebulized with NCs. The confocal microscopy revealed the presence of NCs (red fluorescence) across all lung lobes, including regions with compromised alveolar architecture. Moreover, the biodistribution of the NCs appeared to be homogeneous throughout the lung tissue, as Cy5 fluorescence was consistently detected in multiple lobes of both healthy and ALI animals. Scale bar: 20 μm. Green: membranes stained with Cell Mask; red: NCs (Cy5); 60× magnification; average zoom = 1×. Data is shown as the mean ± sem (*n* = 6). Reproduced from ref 135. Available under a CC-BY 4.0. Copyright 2024 John Wiley and Sons.

## 6 Patents

Interest in the pulmonary delivery of drugs has grown rapidly over the years, as has the search for nanostructured PLGA products. In this sense, products developed with PLGA nanoparticles for the treatment of various lung diseases are of great interest. A search for patents was carried out on the WIPO (World Intellectual Property Organization) and Espacenet platforms with the combination of the terms “PLGA nanoparticles” and “pulmonary delivery” and “inhalable”. No results were found on the WIPO platform. On the other hand, 88 patents were found through Espacenet. All of them were checked individually to select those that met the purposes of this work. In all, 10 patents met the criteria and are described in Table 2.

**Table 2:** Patents on PLGA-Based Nanoparticles for Pulmonary Drug Delivery

patent name	patent number	country	PLGA function	disease	active pharmaceutical ingredient	refs
compositions and methods for treating bacterial infections	WO2021207126A1	United States of America	drug carrier	lung and respiratory tract infections	miRNA and/or antibiotic	^25^
dry powder formulations for mRNA	US2020022921A1	United States of America	surface functionalization	cystic fibrosis	mRNA	^26^
tilmicosin/g-type alginate oligosaccharide aerosol inhalation nano suspension and preparation method thereof	CN117838672A	China	surface functionalization	treatment of respiratory diseases in livestock and poultry	tilmicosin/G-type alginate oligosaccharide	^27^
cationic CaMKII inhibiting nanoparticles for the treatment of allergic asthma	WO2018031771A1	United States of America	drug carrier	asthma	CaMKII inhibitor peptide	^28^
nanocomposites for enhanced cellular payload delivery	WO2024077034A2	United States of America	drug carrier	not specified	nucleic acids, proteins, peptides, chemotherapeutics, vaccine components, antibiotics, or a combination of two or more of the foregoing	^29^
biocompatible nanopolymer particles comprising active ingredients for pulmonary application	US2014127311A1	United States of America	drug carrier	pulmonary hypertension or erectile dysfunction	phosphodiesterase inhibitors (PDE inhibitors) or guanylate cyclase activators or guanylate cyclase stimulators or endothelin receptor antagonists or the prostanoids	^30^
core–shell structure nanoparticles with adjustable and controllable flexibility and preparation method and application of core–shell structure nanoparticles	CN117582419A	China	surface functionalization	not specified	DNA, mRNA or siRNA	^31^
inhalant formulation for treatment of pulmonary diseases and preparation method thereof	KR20140073745A	Republic of Korea	drug carrier	lung diseases, including COPD	tiotropium, ypratropium, glycopyrronium, Indacaterol, formoterol and similars	^32^
aerosol inhalation drug-loaded nanoparticles, siRNA sequence group for treating pulmonary fibrosis and design method of siRNA sequence group	CN116406258B	China	drug carrier	pulmonary fibrosis	siRNA or mRNA	^33^
inhalable pegylated composite nanoparticles of plga and polyethylenimine for delivery of pDNA to lungs	IN3511MU2015A	India	drug carrier	cystic fibrosis	pDNA	^34^

The table shows a variety of patents highlighting the use of PLGA nanoparticles for pulmonary drug delivery, revealing the diversity of applications and functions of this technology. The majority of patents come from the United States (50%), followed by China (30%), with smaller contributions from Korea and India, reflecting a global panorama of innovation in the area. In addition, 70% of the patents were filed by academia,^25,27–32^ 20% by industry^26,33^ and 10% did not specify the origin of the filing.^34^ The large number of applications filed by academia demonstrates the diversity of studies and interest in this area of research PLGA is widely used both as a nanostructured carrier and for functionalizing the surface of nanoparticles, which improves the delivery of specific therapeutic agents.

The patents cover a wide range of compounds, including mRNA, antibiotics and peptides, with applications ranging from the treatment of respiratory infections and pulmonary fibrosis to rare diseases such as cystic fibrosis. The analysis reveals a growing trend toward customizing the surface properties of nanoparticles to improve the specific delivery and controlled release of therapeutic agents. The variety of products, which includes dry formulations and nanoparticles loaded with siRNA, illustrates the versatility of PLGA-based nanoparticles and their potential to transform drug delivery and the treatment of respiratory diseases. This ongoing advance demonstrates not only the innovative capacity of PLGA, but also the growing international effort to research and develop solutions for improving complex treatments through nanotechnology.

Despite extensive research and promising preclinical results demonstrating the potential of PLGA NPs for pulmonary drug delivery, no inhalable pharmaceutical products based on PLGA nanoformulations have yet received regulatory approval or reached the market. This gap primarily reflects several unresolved translational challenges that hinder clinical implementation. Key obstacles include the complexity of scaling up production processes while preserving particle size uniformity, surface properties, and functional performance critical for effective lung deposition. Additionally, ensuring the long-term physicochemical and aerodynamic stability of dry powder formulations remains a significant hurdle, as instability can compromise dose reproducibility and therapeutic efficacy during storage and administration. Moreover, the limited understanding of the chronic pulmonary toxicity and immunogenicity associated with repeated exposure to polymeric NPs further restricts their clinical translation, underscoring the need for comprehensive safety evaluations in relevant animal models and ultimately in humans.^22,134^ Although PLGA-based nanosystems inherently offer numerous advantages—including excellent biocompatibility, biodegradability, and the capacity for sustained and controlled drug release—these benefits must be carefully balanced against potential risks. Consequently, continued research efforts are essential to optimize formulation strategies, develop scalable manufacturing techniques, and thoroughly assess long-term safety profiles to enable the successful integration of PLGA nanocarriers into routine inhalation therapies.

## 7 Conclusions and Future Perspectives

PLGA-based nanoparticles are emerging as a promising strategy for pulmonary drug delivery, offering a number of significant advantages for improving the treatment of respiratory diseases. These nanoparticles are notable for their ability to encapsulate both hydrophobic and hydrophilic drugs, ensuring a controlled and prolonged release of the drug in the respiratory tract. PLGA’s biocompatibility and biodegradability provide safety for clinical use and a reduction in systemic adverse effects, which can increase therapeutic efficacy. In addition, the functionalization of the surface of PLGA nanoparticles makes it possible to extend the therapeutic range and enhance drug delivery to specific targets. These advantages have led to a significant increase in the number of scientific publications and patent filings related to the pulmonary administration of these nanoparticles for the treatment of different pathologies.

However, large-scale production of PLGA nanoparticles can present significant challenges, requiring the development of scalable and economically viable manufacturing methods. The complexity of the production process and the need to guarantee the homogeneity and quality of the nanoparticles represent obstacles that require robust solutions. In this sense, microfluidic technology can facilitate the continuous production and scale-up of PLGA nanoparticles by allowing precise control of the preparation conditions and obtaining homogeneous particles on a large scale, increasing the efficiency and reproducibility of the manufacturing process.

Furthermore, a thorough evaluation of preclinical aspects is crucial to ensure the safety and efficacy of nanoparticles before their application in humans. Further studies are essential to assess the long-term toxicity, stability, pharmacokinetic parameters and efficacy of nanoparticles in different pulmonary conditions.

## Abbreviations

PLGA: poly­(lactic-*co*-glycolic acid)
NPs: nanoparticles
CF: cystic fibrosis
COPD: chronic obstructive pulmonary disease
